# The Dissolving Ability of Different Organic Solvents on Three Different Root Canal Sealers: In Vitro Study

**Published:** 2012-10-13

**Authors:** Mubashir Mushtaq, Ajaz Masoodi, Riyaz Farooq, Fayiza Yaqoob Khan

**Affiliations:** 1. Department of Conservative Dentistry and Endodontics, Government Dental College and Hospital Srinagar, Kashmir, India; 2. Department of Periodontics and Oral Implantology, Government Dental College and Hospital Srinagar, Kashmir, India

**Keywords:** AH Plus, Apexit Plus, Endodontics, Endoflas FS, RC Sealers, Retreatment, Solvents

## Abstract

**Introduction:**

The purpose of this in vitro study was to evaluate three common gutta-percha solvents’ effectiveness in dissolving three different types of root canal sealers.

**Materials and Methods:**

The solubility of three different root canal sealers (AH Plus, Apexit Plus and Endoflas FS) was assessed in xylene, refined orange oil, tetrachloroethylene and distilled water (control). One-hundred twenty samples of root canal sealers were prepared and then divided into three equal groups (n=40). Each group was further divided into four equal subgroups (n=10) for immersion in the respective solvents for a 10 minute immersion period. The mean amount of weight loss was determined for each material in each solvent during the specified immersion period, and the values were subjected to statistical analysis.

**Results:**

Xylene exhibited the greatest dissolving efficacy for AH Plus, followed by refined orange oil and tetrachloroethylene. Xylene was also able to dissolve the greatest amount of Apexit Plus, followed by refined orange oil and tetrachloroethylene which were equally effective in dissolving Apexit Plus. For Endoflas FS, maximum dissolving efficacy was seen with tetrachloroethylene followed by refined orange oil and xylene.

**Conclusion:**

The results showed that xylene, refined orange oil and tetrachloroethylene can be used for the removal of AH Plus, Apexit plus and Endoflas FS sealers during endodontic retreatment. Further clinical investigations are needed to evaluate the efficacy of these solvents on different sealers.

## Introduction

The basic objective of nonsurgical endodontic retreatment is an attempt to re-establish healthy periapical tissues after inefficient treatment or reinfection of an obturated root canal system because of coronal or apical leakage. Access is required to the entire root canal system through removal of the defective root canal filling, further cleaning and shaping and reobturation [1]. Removal of root canal fillings can be conducted with several techniques. These include rotary files, ultrasonic instruments, and hand files in combination with heat or chemicals [2]. Organic solvents have been used to aid removal of gutta-percha and sealer [3][4]. However, all solvents are known to be toxic to the periapical tissues and should be used with caution [5][6]. Laboratory studies have shown the effectiveness of various solvents against different types of endodontic sealers. Chloroform and xylol have been shown to dissolve most root canal filling materials [5][6]. Because of concerns about the carcinogenicity of chloroform, clinicians and researchers have a renewed interest in finding alternative solvents [7][8]. Other solvents available for the dissolution of gutta-percha/sealer are refined orange oil and tetrachloroethylene. There are as yet no studies that show the effectiveness of these solvents on Apexit Plus and Endoflas FS sealers. Hence the purpose of this study was to compare and evaluate the dissolving capabilities of various endodontic solvents such as xylene, tetrachloroethylene and refined orange oil on AH Plus, Apexit Plus and Endoflas FS root canal sealers.

## Materials and Methods

Calcium hydroxide-based/Apexit Plus (Ivoclar Vivadent, Schaan, Liechtenstein), Epoxy-amine resin based/AH Plus (Dentsply, DeTrey, Konstanz, Germany), and zinc oxide eugenol-based/Endoflas FS (Sanlor Laboratories, Cali, Colombia) sealers were used in this in vitro study. The compositions of the different sealers used in the study as given by the manufacturers are summarized in Table 1.

**Table 1:** Composition of the different sealers used in the study as provided by the manufacturers

**Apexit plus**	***Base:*** Calcium hydroxide/Calcium oxide, Hydrated collophonium, fillers and other auxiliary materials (highly dispersed silicon dioxide, phosphoric acid alkyl ester)	***Activator:*** Disalicylate,Bismuth hydroxide/Bismuth carbonate, fillers and other auxiliary materials (highly dispersed silicon dioxide, phosphoric acid alkyl ester)
**AH plus**	***Paste A:*** Bisphenol A epoxy resin, Bisphenol F epoxy resin, Calcium tungstate, Zirconium oxide, Silica, Iron Oxide Pigments	***Paste B:*** Dibenzyldiamine, Amino-adamantane, Tricyclodecane-diamine, Calcium tungstate, Zirconium oxide, Silica, Silicone oil
**Endoflas FS**	***Powder:*** Zinc oxide, Iodoform, Calcium hydroxide, Barium sulfate	***Liquid:*** Eugenol, Accelerator, Zinc acetate

Standardized stainless steel molds 8 mm in diameter and 2 mm in height were used for the preparation of sealer specimens. Sealer cements were mixed in accordance with the manufacturers’ instructions and introduced into the molds. A microscope slide was then pressed onto the upper surface of each mold to make the surface flat. Ten minutes after mixing, the molds were then transferred to a humidifier with 80% relative humidity and 37±1˚C temperature for 72 hours. Then they were removed from the chamber and excess material was trimmed to the surface level of the mold with a scalpel and brush. The samples were weighed in grams (up to four decimal places) on a digital analytical scale (Shimadzu Electronic Weighing Machine, Japan, Model: AEL-200) prior to immersion in the solvent to obtain the initial mass (m1). The weights were recorded in duplicate. One hundred twenty samples of root canal sealers were prepared and then divided into three equal groups (n=40). Each group was further divided into four equal subgroups (n=10) for immersion in the respective solvents. The selected solvents were xylene (Fisher Scientific, Mumbai, India), tetrachloroethylene (Ammdent, India), and refined orange oil (Nipponshika, Yakuhin Co. Ltd, Shimonoseki, Japan). Distilled water (Milli-Q, Millipore Corp., Billerica, MA, USA) served as a control.

Sealer specimens were immersed in 20 mL of solvent stored in an amber glass bottle with a screw cap (Corning Inc., New York, NY, USA) at room temperature. The sealer specimens were immersed in the respective solvents for a 10 minute immersion period. After the specified immersion period, the specimens were removed from the glass vial with the aid of tweezers with silicone tips, rinsed with 100 mL of double-distilled water and then blotted dry with absorbent paper. Samples were allowed to dry in an oven (Thermo Scientific Series 6000, UK) for 24 hours at 37±1˚C and then kept in a dehumidifier/desiccator (SKS Science products, NY, USA) for 15 minutes. Thereafter, they were weighed (m2), and the amount of sealer lost from each specimen was determined was the difference between this measurement and the original weight of the sealer. The means and standard deviations of dissolution (weight loss) in grams were calculated at the specified immersion time interval for each group of specimens (Table 2).

**Table 2:** Means (SD) of dissolution weight loss in grams of the different sealers in the respective test solvents

** **	**Xylene**	**Refined orange oil**	**Tetrachloroethylene**	**Distilled water**
**AH Plus**	0.075 (0.006)	0.057 (0.004)	0.036 (0.002)	0.004 (0.0005)
**Apexit Plus**	0.057 (0.004)	0.035 (0.002)	0.034 (0.002)	0.005 (0.0006)
**Endoflas FS**	0.041 (0.003)	0.051 (0.005)	0.08 (0.007)	0.006 (0.0007)

The values were compared by factorial analysis of variance (ANOVA) using SPSS 16.0 software (SPSS Inc., Chicago, IL, USA), and differences amongst the materials were calculated and multiple comparison tests performed to identify statistically homogenous subgroups (P<0.05) using a post hoc least significant difference test(LSD) with the value of statistical significance set at 0.05.

## Results

The different solvents effect on AH Plus showed there was a significant amount of weight loss in all groups except the control group (P<0.05). Xylene exhibited the best dissolving capability on AH Plus followed by refined orange oil and tetrachloroethylene.

Comparison of the solubilizing effect of different solvents on Apexit Plus showed there was a significant amount of weight loss in all solvents except the control group (P<0.05). This time xylene exhibited the best dissolving capability. Refined orange oil and tetrachloroethylene did not show any significant difference in dissolving Apexit Plus sealer.

The different solvents dissolved significant amount of Endoflas FS in all the groups except the control group (P<0.05). Tetrachloroethylene exhibited the best dissolving efficacy for this sealer followed by refined orange oil and xylene in descending order.

## Discussion

During nonsurgical endodontic retreatment it is important to remove as much sealer and gutta-percha as possible in order to uncover any remnants of necrotic tissue or bacteria which may be responsible for endodontic failure. Thermal, mechanical or chemical methods are used alone or in combination to remove the root canal filling [6]. Using purely mechanical means to remove gutta-percha is problematic because root perforation, canal straightening or alteration of the original canal shape may result. Solvents have been used in the past to soften and dissolve root canal fillings. Solvents available for dissolution of gutta-percha filling material are as follows: Chloroform, Eucalyptol oil, Xylene, Halothane, Turpentine oil, Pandine needle oil. When small, underprepared and curved canals must be negotiated often with solvents and small K-type files.

So we conducted a study to comparatively evaluate the dissolving capabilities of various endodontic solvents such as xylene, tetrachloroethylene and refined orange oil on three different root canal sealers including AH Plus, Apexit Plus and Endoflas FS.

In clinical practice, chloroform is supposedly the most effective and widely used solvent for most root canal filling materials. Other solvents include refined orange oil, halothane, tetrachloroethylene and xylene. No studies have yet evaluated the above solvents to remove Apexit Plus or Endoflas FS sealers or their comparative evaluation with other commonly used sealers. Due to concerns about the carcinogenicity of chloroform, clinicians and researchers have developed a renewed interest in finding alternative solvents. Halothane is a possible alternative solvent to chloroform. It is a fluorinated hydrocarbon used for induction of anesthesia. Halothane, however, is not without drawbacks. Idiosyncratic hepatic necrosis is a potential side effect following repeated use of halothane-induced anesthesia. Idiosyncratic toxicities are a major concern because they are difficult to predict and not usually present until the patient has been previously exposed to the agent. They are also host dependent and dose dependent [9]. The incidence of halothane hepatitis is on the order of one in 10,000 exposures. Other chlorine-containing solvents like tetrachloroethylene are not hepatotoxic [10], so tetrachloroethylene can be considered as a quite safe alternative to chloroform as compared to Halothane.

Chloroform tends to be messy and inconvenient in endodontic retreatment procedures as it dissolves rather than softens the root canal filling material, leaving residues on the walls of the pulp chamber. Its fast evaporation makes it essential to add more and more solvent as soon as it evaporates. Xylene, on the other hand, dissolves root canal filling material more slowly, thus allowing better control and removal of softened rather than liquefied root canal filling material. Softening and mechanical removal of gutta-percha, rather than dissolving it, may prove to be not only efficient but also a biologically safer procedure [11]. This can be accomplished by means of a cotton pellet moistened with a solvent in the chamber and removing the root canal filling at the following appointment. Because the aged root canal filling tends to become harder and more difficult to remove, such a procedure is of potential importance because it softens the root canal filling slowly before any attempt is made to remove it [11]. Essential oil extracted from the peel of sweet orange, citrus aurantium, is easy to obtain and suitable for rapid opening of the root canal, especially in zinc oxide cemented root fillings associated or not with gutta-percha cones. Orange oil is an excellent alternative solvent compared to potentially toxic solvents, being used either on zinc oxide eugenol cement or to soften and dissolve gutta-percha [12]. D-Limonene (Refined orange oil) is found widely in citrus and many other plant species and is a major constituent of many essential oils. It is used extensively as a component in flavorings and fragrances, as a chemical intermediate and in insect repellants. The use of essential oils in endodontics is growing because of their proven safety, biocompatibility and non-carcinogenicity [13].

In this study, 120 samples of root canal sealers were prepared to check their dissolution in xylene, refined orange oil and tetrachloroethylene, and the weight loss in each root canal sealer sample was calculated to check their dissolution. Weight loss varied within the specimens because the solvent contacts only part of the sample material (surface area), thereby dissolving it slowly [5]. Evaluation criteria for the amount of material dissolved were according to Martos et al. [14] and the immersion time of the specimens was in accordance with Whitworth and Boursin [15].

The results of the present study indicate that all of the root canal sealers used in the study were soluble in the test solvents, although there were variations between the groups. This is in accordance with several previous studies [3][16]. In those studies, xylene was reported to be the solvent with the greatest capacity for dissolving most endodontic sealers.

AH26 and AH Plus root canal sealers are resin-based materials. There is little information in the literature to suggest which solvents may be effective on these sealers. Bodrumlu and Kayaoglu, in their in vitro study, concluded that AH Plus dissolved to some extent, and more than Ketac-Endo, using either eucalyptus oil or chloroform as the solvent [17]. Hansen [18] tested chloroform, xylol, eucalyptol and orange oil. A Hedstrom file was used to penetrate the length of tubes (15 mm) obturated with gutta-percha and AH26, procosol or Sealapex after 40 minutes. Only chloroform dissolved AH26 in 40 minutes. Whitworth and Boursin evaluated the dissolution of Tubli-Seal, Apexit and AH Plus sealers in chloroform and halothane and concluded that AH Plus was significantly more soluble than all other materials in both chloroform and halothane [15]. In the present study, xylene exhibited the maximum dissolving efficacy for AH plus, followed by refined orange oil and tetrachloroethylene.

Apexit and its successor Apexit Plus are calcium hydroxide based-sealers. Apexit Plus differs from Apexit in that it is supplied in a more convenient delivery form and has a more hydrophilic formulation. Consequently, the material is more reliable if used in thicker layers. Whitworth and Boursin evaluated the dissolution of Tubli-Seal, Apexit and AH Plus sealers in chloroform and halothane and concluded that Apexit was significantly more soluble in halothane than chloroform [15]. An extensive review of the literature revealed no studies evaluating the dissolution of Apexit Plus sealer in different solvents used during endodontic retreatment. In the present study, xylene exhibited the maximum dissolving efficacy for Apexit Plus. Refined orange oil and tetrachloroethylene dissolved this sealer to the same extent.

Endoflas-FS is a zinc oxide eugenol-based sealer. Zinc oxide eugenol-based sealers have traditionally been the most commonly employed sealants. They have served as the gold standard against which other sealers are compared, as they reasonably meet most of Grossman's requirements for sealers [19]. In order to improve the antimicrobial efficacy of zinc oxide eugenol sealers, known bactericidal agents such as iodoform have been incorporated, resulting in modified zinc oxide eugenol based sealers such as Endoflas FS and Medicated Canal Sealer (MCS) [20]. There is a paucity of studies evaluating the dissolution of Endoflas FS in different organic solvents. In the present study, tetrachloroethylene exhibited the maximum dissolving efficacy for Endoflas FS followed by refined orange oil and xylene in descending order.

## Conclusions

Within the limits of this in vitro investigation, it can be concluded that each sealer may be sensitive to a specific solvent. AH Plus and Apexit Plus dissolved the most with xylene compared with the other solvents.

Tetrachloroethylene was most effective for dissolving Endoflas FS sealer.
